# Epidemiology of neonatal sepsis in two neonatal intensive care units in Krakow, Poland in 2016–2017 years

**DOI:** 10.1186/s12879-023-08836-2

**Published:** 2023-11-24

**Authors:** Edyta Golińska, Ł Kozień, A Tomusiak-Plebanek, J Kędzierska, M Dorycka, R Lauterbach, D Pawlik, B Rzepecka-Węglarz, M Janiszewska, PB Heczko, J Wojkowska-Mach, M Strus

**Affiliations:** 1 Chair of Microbiology, Jagiellonian University Medical College, Czysta 18 Street, Cracow, 31-121 Poland; 2 Department of Microbiology, University Hospital, Cracow, Poland; 3 Microbiological Laboratory, Diagnostics Inc. Krakow Branch, Cracow, Poland; 4 Department of Neonatology, Medical College, Jagiellonian University, Cracow, Poland; 5 Department of Neonatal Intensive Care, “UJASTEK” Medical Centre, Cracow, Poland; 6 Department of Informatics and Medical Statistics with E-learning Laboratory, Medical University, Lublin, Poland

**Keywords:** Sepsis, Neonates, Neonatal intensive care, *E.coli*, Coagulase-negative staphylococci

## Abstract

**Background:**

Sepsis in low-birth-weight neonates remains one of the most significant causes of neonatal morbidity and mortality. Approximately 3 million newborns suffer from sepsis globally every year. The aim of this study was to compare demographic and clinical features, as well as etiology and antibiotic susceptibility, of the main pathogens related to neonatal sepsis in two neonatal intensive units during a two-year period.

**Methods:**

We observed early-onset (EO-BSI) and late-onset bloodstream infections (LO-BSI) cases in two high-reference neonatal intensive care units (NICU) over a 24-month period (2016–2017). Samples of patients’ blood were tested for the presence of the microorganisms. All bacterial isolates were tested for susceptibility to antibiotics.

**Results:**

The majority of sepsis cases weighed above 1000 g and were born by cesarean section. About 10% of the EO-BSI group died. There were differences in the EO-BSI /LO-BSI ratio in the compared wards due to differences among the admitted children. The most common pathogens isolated from blood were coagulase-negative staphylococci (CoNS) were represented by two dominating species: *S. epidermidis* and *S. haemolyticus*, followed by *Klebsiella spp*. strains and *E.coli*, which were mostly found in EO-BSI cases. No single *S. agalactiae* (GBS) strain was isolated. The majority of CoNS strains were resistant to methicillin, half were resistant to aminoglycosides, and one-third were resistant to macrolides and lincosamides. Half of the Gram-negative rods were resistant to beta-lactams.

**Conclusions:**

The epidemiology of sepsis in two observed NICUs is comparable to data obtained from other studies with a predominance of methicillin-resistant CoNS in LO-BSI and beta-lactam resistant *E. coli* in EO-BSI. It is of importance that the campaign for controlling GBS carriage in pregnant women in Poland resulted in the disappearance of GBS as a cause of sepsis. Unfortunately, there are no such measures to control *E.coli* related sepsis.

## Introduction

Neonatal sepsis is one of the major causes of mortality in neonates and despite recent medical advancements, still remains a significant global health challenge. It is estimated that 3 million newborns suffer from sepsis globally every year [1]. Based on the timing of onset of the infection and presumed mode of transmission, neonatal sepsis is commonly divided into early-onset sepsis, EO-BSI (occurring in the first 72 h and predominantly associated with microorganisms transferred from the mother), and late-onset sepsis, LO-BSI (occurring after 72 h of postnatal life and usually associated with nosocomial or community-acquired infection) [2].

Over the last century, the spectrum of pathogens causing neonatal sepsis has changed [3–5]. Recent reviews revealed that coagulase-negative staphylococci (CoNS) are a common cause of EO-BSI and the main causative agents of LO-BSI (mainly in very-low-birth-weight infants), with its proportion ranging from 30 to 60% [6, 7]. This change is related to the rapidly increasing usage of invasive medical devices, like central venous catheters. When inserted into vessels it enables common colonizers of the human skin and mucous membranes, mostly CoNS, to allow bloodstream invasion and become an important source of sepsis [6]. Moreover, over the last few decades most of the important pathogens related to sepsis, including CoNS, have developed resistance to multiple antibiotics which results in limited treatment options and contributes to a significant health and economic burden [8, 9]. As a result, bloodstream infections in infants, especially in low- or very low birth weight neonates, are associated with significant morbidity and mortality [10].

The increasing antimicrobial resistance and mortality in neonatal intensive care units create an urgency in the understanding of the epidemiology and risk factors of neonatal sepsis. Therefore, the aim of this study was to compare demographic and clinical features, as well as etiology and antibiotic susceptibility, of the main pathogens related to neonatal sepsis in two neonatal intensive units during a two-year period.

## Materials and methods

### Study population

This was a retrospective cross-sectional observation study in two neonatal intensive care units in Krakow, Poland, in 2016–2017. A total of 311 neonates hospitalized in the Clinic of Neonatology, University Hospital, Jagiellonian University Medical College (Ward A) and Neonatal Ward at Ujastek Hospital in Cracow (Ward B) with laboratory-confirmed bloodstream infections were included. In Ward B, only children born in the same district gynecology and obstetrics hospital were hospitalized, while Ward A serves as a regional neonatology center for the university hospital and neonates with more complicated illnesses, usually older than 48 h, are transferred to this ward from other hospitals where they had been born. During 2016 and 2017 6,682 children were born at the University Hospital and 5,345 at the Ujastek Hospital.

Case patients were defined according to Gastmeier et al. [11], for neonates with birth weights < 1.5 kg. EO-BSI was defined as infection onset in less than 72 h after birth whereas late-onset bloodstream infections (LO-BSI) when diagnose > 72 h after delivery [11]. Infants with more than one episode case of BSI were included in the study and regarded as separate cases; thus, the number of cases was higher than the number of newborns: altogether there were 32 newborns, with 10% having more than one episode of BSI. The Repeat Infection Timeframe (RIT) was a 14-day timeframe during which no new infections of the same type were reported (CDC, Identifying Healthcare-associated Infections (HAI) for NHSN Surveillance; https://www.cdc.gov/nhsn/PDFs/pscManual/2PSC_IdentifyingHAIs_NHSNcurrent.pdf ). All cases of EO-BSI were due to a single etiological factor. Data not available included the gender of two newborns and the birth weight of 18 patients.

### Microbiological cultures

Simultaneously with the laboratory diagnostic tests, samples of blood were taken for culture. Aseptically taken blood specimens were injected into an aerobic blood culture bottle (Bactec Plus 26 Aerobic; BD Microbiology Systems), incubated, and then subcultured on MacConkey Agar (Oxoid, UK), Columbia Blood Agar (Oxoid, UK) at 37 °C for 24 h and Sabouraud Agar (Oxoid, UK) at 37 °C for 36 h. No growth in the culture media (after 7 days of incubation) indicated a negative result. Isolation of the microorganism(s) from at least one blood culture was defined as a bloodstream infection. All cultured microorganisms were identified using mass spectrometry (MALDI Biotyper, Bruker Billerica, MA, USA) according to the manufacturer’s instructions. Only strains from laboratory-confirmed bloodstream infections of newborns with clinical signs of BSI were analyzed according to the Gastmeier definition [11].

This study utilizes the terms “other Gram-positive cocci” and “other Gram-negative rods” meaning: Other Gram-positive cocci: *Enterococcus faecalis, Enterococcus faecium*, viridans streptococci group, *Streptococcus sanguis, Streptococcus oralis;* Other Gram-negative rods: *Serratia marcescens, Enterobacter cloacae, Proteus mirabilis, Acinetobacter baumani, Serratia liquefaciens, Pseudomonas aeruginosa.*

### Antimicrobial susceptibility test

All bacterial isolates were tested for susceptibility to antibiotics using the manual disc diffusion method according to the European Committee on Antimicrobial Susceptibility Testing (EUCAST) standards. The inhibition zones were also measured and interpreted according to the EUCAST recommendations (https://www.eucast.org/fileadmin/src/media/PDFs/EUCAST_files/Breakpoint_tables/v_6.0_Breakpoint_table.pdf). E-tests were used for vancomycin and teicoplanin resistance testing (bioMérieux, Paris, France).

The methicillin-resistant coagulase-negative *Staphylococcus* (MRCNS) resistance phenotype was detected using a cefoxitin disc (30 μg) according to the EUCAST guidelines. The macrolide-lincosamide (MLS) resistance phenotype of the isolates was determined according to a previously published protocol [12].

Extended-spectrum beta-lactamases (ESBL) activity was detected with a modified double disk synergy test using a combination of cefotaxime, ceftazidime, cefepime, and aztreonam discs, placed 20 mm apart around a disc containing amoxicillin/clavulanic acid [13].

### Statistical methods

The Fisher exact test and the Pearson chi-square test were used to examine the relationships between the nominal variables. A post-hoc test was applied to calculate the significance and strength of the relationship between individual pairs of groups in the case of nominal multilevel variables. The relationships between the nominal dichotomous and continuous variables were examined using the point-biserial correlation. The examination of the differences in means for continuous variables was selected based on the results of the Shapiro-Wilk normality test. In the case of non-normal distributions of two independent samples on continuous scales, the Mann-Whitney U test was used.

The statistical calculations were performed by the R v.4.1.1 statistical computing environment, IDE RStudio v. 1.4.1717 (R Core Team, 2021.). The significance level of the statistical tests was set at α = 0.05.

## Results

### Demographic and clinical characteristics of the study population

The total number of cases of neonatal sepsis was 349 (156 female newborns and 193 male newborns) in 311 patients (Table 1). The median birthweight was 1245 g (1st quartile (Q1) 855 g; 2nd quartile (Q3) 1700 g). The mean values of the neonatal weight distribution for EO-BSI cases differed significantly from the mean values of the neonatal weight distribution in LO-BSI cases (*p* = 0.022). Children with a birthweight less than 750 g constituted 16.4% of the cases, nine of the children were triplets and 81 were twins (the number is odd because both twins did not always develop sepsis).

The majority of the patients (271 newborns, 87.1%) were born by cesarean section (CS), with no significant difference between EO-BSI and LO-BSI. No differences were found in the group of newborns with EO-BSI and LO-BSI in terms of gender, multiple births, and the early or late occurrence of sepsis. There was, however, an association between the occurrence of EO-BSI / LO-BSI and the department where a child was treated (*p* = 0.011) (Table 1). The fatality case rate of neonatal sepsis was 10.3% (n = 36) with no significant difference between EO-BSI and LO-BSI.

**Table 1:** Characteristics of the neonates with EO-BSI and LO-BSI

Newborns included in the study		Total (n = 349)	EO-BSI cases(n = 29)	LO-BSI cases(n = 320)	*p*-value	
Birth weight Med (Q1;Q3) [grams]		1245 (855; 1700)	1500 (1100; 2640)	1240 (840; 1675)	0.022	
Cesarean section [n/%]	Yes	271 (82.1%)	24 (82.2%)	247 (82.1%)	1.000	
	No	59 (17.9%)	5 (17.2%)	54 (17.9%)		
Gender [n/%]	Female	155 (44.7%)	12 (41.4%)	143 (45.0%)	0.845	
	Male	192 (55.3%)	17 (58.6%)	175 (55.0%)		
Multiple births [n/%]	Yes	90 (25.8%)	5 17.2%)	85 (26.6%)	0.277	
	No	259 (74.2%)	24 (82.8%)	235 (73.4%)		
Ward [n/%]	A	300 (86.0%)	20 (69.0%)	280 (87.5%)	0.011	
	B	49 (14.0%)	9 (31.0%)	40 (12.5%)		
Death of the patient during hospitalization [n/%]	Yes	36 (10.3%)	6 (20.7%)	30 (9.4%)	0.11	
	No	313 (89.7%)	23 (79.7%)	290 (90.6%)		

Legend: Med = Median; Q = Interquartile range; Ward A = Clinic of Neonatology, University Hospital, Jagiellonian University Medical College; Ward B = Neonatal Ward at Ujastek Hospital in Cracow

The incidence rate of EO-BSI was 2.2% (n = 29). The median birthweight in this group was 1500 g (Q1 = 1100 g; Q3 = 2640 g). LO-BSI was diagnosed in 320 newborns, the incidence rate was 24.9%. The median birthweight in this group was 1240 g (Q1 = 840 g; Q3 = 1675 g).

### Etiologic factors related to bloodstream infection

Coagulase-negative staphylococci were the most commonly isolated group of bacteria in both EO-BSI and LO-BSI groups. Altogether, 280 (80.3%) CoNS strains were isolated. Among them, isolates classified to *S. epidermidis* species predominated over *S. haemolyticus, S. capitis*, and *S. hominis*, and only a few isolates belonged to other species. Other microorganisms isolated from blood samples were represented by 64 Gram-negative rods, 11 *Enterococcus* spp., 10 *S. aureus*, 3 *Streptococcus* spp., 2 *Candida* spp., and 8 different bacteria. The most common Gram negative rods belonged to the species *Klebsiella* spp. and *E. coli*. In nine blood samples (2.6%) no growth was recorded.

The most frequent bacteria isolated from EO-BSI were CoNS (48.3%) among which *S. hominis* dominated. The main group of bacteria isolated from cases of LO-BSI were also CoNS (83.1%) with the *S. epidermidis* species being the most dominant. In 27 cases of the LO-BSI, multiple pathogenic bacteria were isolated together. Most commonly, *S. epidermidis* strains occurred together with *S. haemolyticus* (Table 2). Significantly more often *E.coli* rods were isolated in the EO-BSI than LO-BSI cases (*p* = 0.001), other genera or species were isolated in both groups with a similar proportion.

**Table 2:** Prevalence of microorganisms cultured from blood of EO-BSI versus LO-BSI cases

Bacterial etiology of sepsis	Total (n = 349)		EO-BSI cases (n = 29)		LO-BSI cases (n = 320)	
		%		%		%
**Coagulase-negative staphylococci**	280	80.2	14	48,3	266	83,1
*S.epidermidis*	131	37.5	2	6.9	129	40.3
*S. haemolyticus*	71	20.3	2	6.9	69	21.6
*S. capitis*	32	9.2	1	3.4	31	9.7
*S. hominis*	18	5.2	4	13.8	14	4.4
*S. lugdunensis*	2	0.6	0	0	2	0.6
*S. warneri*	2	0.6	1	3.4	1	0.3
*S. xylosus*	1	0.3	1	3.4	0	0
*S. caprae*	1	0.3	1	3.4	0	0
CoNS	22	6.3	2	6.9	20	6.3
**Staphylococcus aureus**	**10**	**2.9**	**2**	**6.9**	**8**	**2.5**
**Streptococcus spp.**	**3**	**0.9**	**2**	**6.9**	**1**	**0.3**
*S. viridans*	1	0.3	0	0	1	0.3
*S. sanguinis*	1	0.3	1	3.4	0	0
*S. oralis*	1	0.3	1	3.4	0	0
**Enterococcus spp.**	**11**	**3.2**	**1**	3.4	**10**	3.1
*E. faecalis*	10	2.9	1	3.4	9	2.8
*E. faecium*	1	0.3	0	0	1	0.3
**Gram negative rods**	**64**	**18.3**	**8**	**27.6**	**56**	**17.5**
*Klebsiella spp.*	32	9.2	2	6.9	30	9.4
*E. coli*	13	3.7	6	20.7	7	2.2
*S. marcescens*	7	2	0	0	7	2.2
*E. cloacae*	6	1.7	0	0	6	1.9
*P. mirabilis*	3	0.9	0	0	3	0.9
*A. baumannii*	1	0.3	0	0	1	0.3
*S. liquefaciens*	1	0.3	0	0	1	0.3
*P. aeruginosa*	1	0.3	0	0	1	0.3
**Other**	**8**	**2.3**	**2**	**6.9**	**6**	**1.9**
**No culture**	**9**	**2.6**	**0**	**0**	**9**	**2.8**
**Mixed**	**27**	**7.7**	**0**	**0**	**27**	**8.4**

Legend: CoNS = coagulase-negative staphylococci: staphylococcal isolates of unknown species. *Klebsiella spp – K.oxytoca, K.pneumoniae, Klebsiella spp.;* Mixed cultures were represented by two coagulase-negative staphylococcal species, mostly *S.epidermidis* and *S.haemolyticus.*

### Antibiotic susceptibility

The prevalence of multidrug-resistant microorganisms (MDR) varied with diagnosis. EO-BSI and LO-BSI were only observed in MRCNS, 60% and 92.4% (0.0001), respectively. In MLSB, 31.2% in EO-BSI and 11.0% in LO-BSI (Table 3).

In the case of Gram-negative rods, the ESBL mechanism was observed in 25% of EO-BSI and 19,6% in strains isolated to LO-BSI.

High-level aminoglycoside resistance mechanism (HLAR) was present in only 10% of enterococcal strains from EO-BSI.

**Table 3:** Resistance mechanisms observed in selected bacteria isolated from EO-BSI and LO-BSI cases

Resistance mechanism n(%)	Number of cases	Total (n = 349)	EO-BSI cases(n = 29)	LO-BSI cases(n = 320)	*p*-value
MRCNS	Yes	227 (90.4%)	9 (60.0%)	218 (92.4%)	0.001
	No	24 (9.6%)	6 (40.0%)	18 (7.6%)	
MLSB	Yes	31 (12.3%)	5 (31.2%)	26 (11.0%)	0.033
	No	221 (87.7%)	11 (68.8%)	210 (89.0%)	
ESBL	Yes	12 (20.3%)	2 (25.0%)	10 (19.6%)	0.66
	No	47 (79.7%)	6 (75.0%)	41 (80.4%)	
HLAR	Yes	1 (8.3%)	0 (0.0%)	1 (10.0%)	1
	No	11 (91.7%)	2 (100.0%)	9 (90.0%)	

Legend: MRCNS = methicillin resistance among coagulase-negative staphylococci; MLSB = macrolide-lincosamide-streptogramin B resistance among CoNS; ESBL = extended-spectrum beta-lactamases among *E.coli, Klebsiella spp*.; HLAR = high-level aminoglycoside resistance among *Enterococcus spp*

### Multivariate prediction analysis

The multivariate of the elements associated with EO-BSI vs. LO-BSI and their etiology demonstrate that *E.coli* significantly caused early sepsis than late sepsis (Odds ratio (OR) 0.5; 95% confidence interval (CI) 0.02; 0.12) (Table 4). Similar results were obtained in the case of Gram-positive bacteria other than CoNS; they also caused early sepsis significantly more often than late sepsis (OR 0.2; 95%CI 0.08; 0.40). Moreover, the analysis confirmed that death was more frequently attributed to early than late onset of sepsis (OR 0.31; 95%CI 0.15; 0.63). The influence of birth weight, despite the statistical significance, is very small (OR 0.9; 95%CI 0.9; 0.9]).

**Table 4:** Multivariate prediction analysis of relations between etiology and onset of sepsis

	*OR*	*z*	*Pr(>|z|)*	*95%CI*
(Intercept)	11.781	7.98	**< 0.001**	6.538; 22.031
Weight	0.999	-5.22	**< 0.001**	0.999; 0.999
*E. coli*	0.500	-6.37	**< 0.001**	0.018; 0.118
Other Gram-positive	0.185	-4.15	**< 0.001**	0.081; 0.404
Death	0.310	-3.24	**< 0.001**	0.151; 0.629

Legend: OR = odds ratio; z = the regression coefficient divided by standard error; Pr(>|z|) = *p*-value associated with the z-value; 95%CI = 95% confidence interval)

## Discussion

The bloodstream infection (BSI), clinically classified as sepsis, is the most frequently observed infection in neonatal wards. According to a European point prevalence survey (2011–2012, based on European Center for Disease Prevention and Control (ECDC) methodology), BSI accounts for 44.6% of all healthcare-associated infections (HAIs). The increasing proportion of sepsis cases among hospitalized neonates is because of the progression of the survival rate of newborns with very low birth weight (VLBW) in modern neonatal intensive care units (NICU). This situation is invariably associated with a high incidence of both early and late infections [14]. It is of interest that in both NICUs studied, mean birth weight of the neonates was relatively high and was not correlated with death.

Different disease burden data have been reported in various studies across multiple NICUs in high-income countries [14]. These local differences are related to the source of the pathogen: an in-utero infection, acquisition from maternal flora, or postnatal acquisition from the hospital or community. The timing of exposure, inoculum size, immune status of the infant, and virulence of the causative agent also influences neonatal sepsis epidemiology in individual centers [15]. Our data are consistent with reports from many other NICUs [14] but there were some local specificities revealed in this study worthy of attention. There were more EO-BSI in Ward B than in Ward A. Although both wards belong to the highest-grade reference centers, there are some differences between them that influence their epidemiological characteristics. In Ward B, only children born in the same district gynecology and obstetrics hospital were hospitalized which explains more EO-BSI. While Ward A serves as a regional neonatology center for the university hospital and neonates with more complicated illnesses, usually older than 48 h, are transferred to this ward from other hospitals where they had been born.

As expected, CoNS dominated over other causes of both EO-BSI and LO-BSI, but they were more numerous in LO-BSI. There were striking differences in the distribution of *S. epidermidis* and *S. haemolyticus* in LO-BSI versus *S. hominis* in EO-BSI. Also, there were differences in CoNS strains resistant to methicillin, and also macrolides/clindamycin isolated from EO-BSI versus LO-BSI cases. These may indicate an occurrence of the epidemic strains of these species in both wards. Independently from this study but in the same time period, selected strains of *S. epidermidis* from LO-BSI cases hospitalized in ward B were MLST typed and sequenced [16]. It appeared that strains belonging to ST29 and ST5 types were found (unpublished). Strains of both clonal types are present as epidemic strains in neonatal ICUs [17]. We also reported recently that CoNS strains related to neonatal sepsis are present in gut microbiota of the premature neonates in one of the wards investigated in this study [18].

Presence of the resistant *Klebsiella* spp. and *Proteus* spp. strains may also be related to their transfer among children inside wards. Among Gram-negative pathogens, *Klebsiella pneumoniae* is one of the most common and dangerous causes of nosocomial infections, especially in NICUs [19]. In Europe, individual cases or small outbreaks of ESBL-producing *Klebsiella* have been reported in several countries (ECDC 2016) [20].

Streptococci grouped together as other Gram-positive cocci were found more frequently in EO-BSI. No single isolate of *S. agalactiae* (GBS) was found, which is contrary to the latest multicenter study on EO-BSI in the United States, based on data collected in [19] centers that showed group B streptococcus as the second most common pathogen after *E.coli* [21]. This discrepancy may indicate that GBS antenatal screening and intrapartum antibiotic prophylaxis recommended in Poland since 2008 [22] is effective. No serious epidemiological studies on GBS- caused sepsis were performed and reported in Poland before introduction of the antibiotic prophylaxis to make a comparison. However, our group made a multicenter study on EO-BSI in Polish NICUs one year after and found *Streptococcus agalactiae* as a cause of 20% of the EOS cases [23]. On the other hand, enterococci were isolated mostly from LO-BSI cases, since they are also members of the neonatal gut microbiota they may persist for longer period of time in the ward and may be transferred among children and personnel [24]. Gram-negative rods, and especially *E.coli*, were more numerous in EO-BSI. This was not the case with *Klebsiella* spp. which were found in EO-BSI and LO-BSI in equal numbers.

*E.coli* is considered the most common cause of death associated with antibiotic resistance (AMR) [25] and the most important pathogen in EO-BSI [21]. Indeed, *E.coli* in this study predominated over other pathogens in EO-BSI cases which is related to chorioamnionitis and also preterm rupture of membranes (ROM) [26]. About one-third of *E.coli* isolates in our study showed the ESBL mechanism of resistance and no one was resistant to the third generation of cephalosporins.

Although we have had no access to data related to the mothers’ pregnancies and labors, our findings support recommendations on the necessity of empirical antibiotic administration for infants born after preterm labor, preterm ROM, or chorioamnionitis [27]. Looking at the gathered data on the occurrence of the multiple resistant pathogens isolated from the neonates’ blood, it may be concluded that among Gram-negative rods only carbapenems were not included in the commonly present resistance mechanisms i.e. no isolate was resistant to them. Therefore, antibiotics of this group may be recommended for treatment in both early- as well late-onset sepsis in the two observed NICUs. On the other hand, the high frequency of methicillin-resistant CoNS in the blood of the late cases may also suggest the use of the cephalosporin III and IV generation, vancomycin, and linezolid in the empirical treatment of LO-BSI cases since [28]. Our study had limitations shared by nearly all studies performed on low- and very-low birth weight neonates of the observed patients and immaturity of their systems. Due to a very low blood volume taken from them for culture it is practically impossible to inoculate multiple bottles with different culture media. Therefore, we were unable to make in-depth study on a full spectrum of the microbes present in the neonates’ blood which could not be recovered due to limited volumes of the blood samples and technical problems related to this like as the examples are strict anaerobes, slow growing and fastidious organisms.

## Conclusion

Neonatal sepsis cases in both NICUs were mostly diagnosed in newborns weighing above 1000 g born by cesarean section. About 10% of them, mostly in the EO-BSI group died during hospitalization. There were differences in the EO-BSI/LO-BSI ratio in the two wards due to the different populations of the admitted children.

This study confirmed that sepsis cases in the compared NICUs were mostly caused by CoNS strains, some of them belonging to known epidemic strains. The majority of the strains isolated from LO-BSI but not EO-BSI cases were resistant to methicillin which also may suggest their epidemic spread in wards. On the other hand, about half of CoNS were resistant to aminoglycosides and one-third to macrolides, lincosamides, and streptogramin. *Klebsiella* strains which were the second cause of both EO-BSI and LO-BSI were 50% resistant to beta-lactams. The same was found in relation to *E.coli* strains which were mostly attributed to EO-BSI.

It should be stressed that no single strain of GBS was isolated from the cases.

Based on these observations, carbapenems and vancomycin are suggested for empirical treatment of both EO-BSI and LO-BSI cases in these NICUs.

## Data Availability

All data generated or analysed during this study are included in this published article.

## References

[1] Fleischmann-Struzek C, Goldfarb DM, Schlattmann P (2018). The global burden of paediatric and neonatal sepsis: a systematic review. Lancet Respir Med.

[2] Cortese F, Scicchitano P, Gesualdo M (2016). Early and late Infections in newborns: where do we stand? A review. Pediatr Neonatol.

[3] McCracken GH (1973). Group B Streptococci: the new challenge in neonatal Infections. J Pediatr J Pediatr.

[4] Jones B, Peake K, Morris AJ et al. 558 Escherichia Coli: a growing problem in early onset neonatal Sepsis. 2004;44. 10.1111/j.1479-828X.2004.00304.x 15598297

[5] Cordero L, Rau R, Taylor D (2004). Enteric gram-negative bacilli bloodstream Infections: 17 years’ experience in a neonatal intensive care unit. Am J Infect Control.

[6] Marchant EA, Boyce GK, Sadarangani M et al. Neonatal sepsis due to coagulase-negative staphylococci. Clin Dev Immunol. 2013;2013. 10.1155/2013/586076 PMC3674645 23762094

[7] Blanchard AC, Quach C, Autmizguine J (2015). Staphylococcal Infections in infants: updates and current challenges. Clin Perinatol.

[8] Xu Z, Shah HN, Misra R et al. The prevalence, antibiotic resistance and mecA characterization of coagulase negative staphylococci recovered from non-healthcare settings in London, UK. Antimicrob Resist Infect Control. 2018;7(1). 10.1186/s13756-018-0367-4 PMC6000976 29946448

[9] Michels R, Last K, Becker SL (2021). Update on coagulase-negative Staphylococci—what the Clinician should know. Microorganisms.

[10] Dong Y, Speer CP (2014). The role of Staphylococcus epidermidis in neonatal sepsis: guarding angel or pathogenic devil?. Int J Med Microbiol.

[11] Gastmeier P, Geffers C, Schwab F (2004). Development of a surveillance system for nosocomial Infections: the component for neonatal intensive care units in Germany. Int J Med Microbiol.

[12] Leclercq R (2002). Mechanisms of resistance to macrolides and lincosamides: Nature of the resistance elements and their clinical implications. Clin Infect Dis.

[13] Drieux L, Brossier F, Sougakoff W (2008). Phenotypic detection of extended-spectrum β-lactamase production in Enterobacteriaceae: review and bench guide. Clin Microbiol Infect.

[14] Wójkowska-Mach, Chmielarczyk S (2019). Neonate Bloodstream Infections in Organization for Economic Cooperation and Development Countries: an update on Epidemiology and Prevention. J Clin Med.

[15] Shane AL, Sánchez PJ, Stoll BJ (2017). Neonatal sepsis. The Lancet.

[16] Thomas JC, Vargas MR, Miragaia M (2007). Improved multilocus sequence typing scheme for Staphylococcus epidermidis. J Clin Microbiol.

[17] Mendes RE, Deshpande LM, Costello AJ, Farrell DJ (2012). Molecular epidemiology of Staphylococcus epidermidis clinical isolates from U.S. hospitals. Antimicrob Agents Chemother.

[18] Golińska E, Strus M, Tomusiak-Plebanek A (2020). Coagulase-negative staphylococci contained in gut microbiota as a primary source of sepsis in low-and very low birth weight neonates. J Clin Med.

[19] Corbella M, Caltagirone M, Gaiarsa S (2018). Characterization of an outbreak of extended-spectrum β-Lactamase-producing klebsiella pneumoniae in a neonatal intensive care unit in Italy. Microb Drug Resist.

[20] European Centre for Disease Prevention and Control (ECDC). European Antimicrobial Resistance Surveillance Network (EARS-Net) database. Summary of the latest data on antibiotic resistance in the European Union EARS-Net surveillance data.

[21] Stoll BJ, Puopolo KM, Hansen NI et al. Early-Onset neonatal Sepsis 2015 to 2017, the rise of Escherichia coli, and the need for Novel Prevention Strategies. JAMA Pediatr. 2020;174(7). 10.1001/jamapediatrics.2020.0593 PMC7199167 32364598

[22] Kotarski J, Heczko PB, Lauterbach R (2008). [Polish Gynecological Society’s recommendations regarding diagnosis and prevention of streptococcus agalactiae Infection in pregnant women and newborns]. Ginekol Pol.

[23] Wójkowska-Mach J, Borszewska-Kornacka M, Domańska J, Gadzinowski J, Gulczyńska E, Helwich E, Kordek A, Pawlik D, Szczapa J, Klamka J, Heczko PB (2012). Early-onset Infections of very-low-birth-weight infants in Polish neonatal intensive care units. Pediatr Infect Dis J.

[24] Pham JT (2020). Challenges of Vancomycin dosing and therapeutic monitoring in neonates. J Pediatr Pharmacol Ther.

[25] Murray CJ, Ikuta KS, Sharara F (2022). Global burden of bacterial antimicrobial resistance in 2019: a systematic analysis. The Lancet.

[26] Newton ER (1993). Chorioamnionitis and intraamniotic Infection. Clin Obstet Gynecol.

[27] Puopolo KM, Benitz WE, Zaoutis TE (2018). Management of neonates born at ≤ 34 6/7 weeks’ Gestation with suspected or proven early-onset bacterial Sepsis. Pediatrics.

[28] Michels R, Last K, Becker SL, Papan C (2021). Update on coagulase-negative staphylococci-what the Clinician should know. Microorganisms.

